# Rare internal malignancies in xeroderma pigmentosum: A report of two cases from Tunisia and analysis of driver mutations

**DOI:** 10.1016/j.cpt.2026.01.003

**Published:** 2026-01-21

**Authors:** Soumaya Rammeh, Yasmine Ben Taher, Mariem Ben Rekaya, Sarra Ben Rejeb, Ahlem Lahmar, Meriem Jones, Faten Zeglaoui, Neila Belguith

**Affiliations:** a Faculty of Medicine of Tunis, LR23ES02, University Tunis El Manar, Tunis 1007, Tunisia; b Pathology Department, Charles Nicolle Hospital, Tunis 1006, Tunisia; c Faculty of Sciences of Bizerte, Carthage University, Bizerte 7021, Tunisia; d Pathology Department, Security Forces Hospital, La Marsa 2078, Tunisia; e Department of Pathology, Mongi Slim Hospital, La Marsa 2070, Tunisia; f Department of Dermatology, Charles Nicolle Hospital, Tunis 1006, Tunisia; g Laboratory of Human Molecular Genetics, Medical School of Sfax, University of Sfax, Sfax 3029, Tunisia; h Department of Congenital and Hereditary Diseases, Charles Nicolle Hospital, Tunis 1007, Tunisia

**Keywords:** Xeroderma pigmentosum, Neoplasms, Somatic mutation, High-throughput nucleotide sequencing

## Abstract

Xeroderma pigmentosum (XP)-associated internal malignancies are characterized by their rarity, early onset, atypical histological presentations, and poorly characterized genomic landscapes. In this study, we report the clinical, pathological, and molecular features of two rare XP-associated internal malignancies, focusing on their somatic mutation profiles. These tumors represented rare histological variants at their respective anatomical sites: an ovarian high-grade sex cord-stromal tumor (HG-SCST) with heterologous rhabdomyosarcomatous differentiation diagnosed in an 18-year-old female, harboring a homozygous XP complementation group C (*XPC*): NM_004628.5:c.1643_1644delTG (p.Val548Alafs∗25) mutation and a renal leiomyosarcoma diagnosed in a 14-year-old male carrying a homozygous *XPC*: NM_004628.5:c.850G>T (p.Glu284∗) mutation. Neither patient had a documented history of cutaneous malignancies. The patient with the ovarian tumor exhibited no response to chemotherapy and succumbed six months after diagnosis, whereas the patient with the renal leiomyosarcoma initially achieved a complete response but subsequently relapsed and died after five years and eight months of follow-up. The literature review identified only one previously reported case of renal leiomyosarcoma in a patient with XP and seven cases of XP-associated malignant ovarian tumors, including four SCSTs. In this study, targeted next-generation sequencing using the AmpliSeq for Illumina Cancer HotSpot Panel identified pathogenic mutations in canonical cancer driver genes: tumor protein p53 (*TP53*) NM_000546.6:c.730G>T (p.Gly244Cys) and platelet-derived growth factor receptor alpha (*PDGFRA*) NM_006206.6:c.2525A>T (p.Asp842Val) mutations in renal leiomyosarcoma and NRAS proto-oncogene, GTPase (*NRAS*) NM_002524.5:c.35G>T (p.Gly12Val) mutation in the ovarian tumor. These findings suggest a potential benefit of personalized therapies for XP patients with internal malignancies.

## Highlight

• Uncommon tumors in two Tunisian xeroderma pigmentosum complementation group C patients: a high-grade ovarian sex cord-stromal tumor and a renal leiomyosarcoma.
• These tumors exhibit early onset and display atypical histological features compared with their sporadic counterparts.
• Tumor protein p53 (*TP53*) p.G244C and platelet-derived growth factor receptor alpha (*PDGFRA*) p.D842V mutations were detected in the renal leiomyosarcoma.
• NRAS proto-oncogene, GTPase (*NRAS*) p.Gly12Val mutation was identified in the ovarian high-grade sex cord-stromal tumor
• Although neither patient developed skin cancer, both succumbed to internal tumors, underscoring the importance of screening for such malignancies.

## Introduction

Xeroderma pigmentosum (XP) represents a paradigmatic example of the interplay between defective DNA repair and cancer development. It results from biallelic pathogenic variants in nucleotide excision repair (NER) genes, leading to genomic instability and marked cancer susceptibility, especially in sun-exposed tissues. Improved diagnosis and preventive care have extended XP patient survival, revealing a distinct oncologic phenotype: a high risk of early-onset atypical internal malignancies.^1^ Due to their scarcity and heterogeneity, limited data exist regarding specific genetic alterations in XP-associated internal malignancies and their potential therapeutic relevance. This knowledge gap poses a particular challenge for XP patients, who are often intolerant to conventional DNA-damaging treatments.

This study reports the clinical, pathological, and molecular features of two rare internal malignancies in two Tunisian XP complementation group C (XPC) patients, focusing on their somatic mutation profiles, and reviews similar cases reported in the literature.

## Cases presentation

Two unrelated XPC patients diagnosed with a renal leiomyosarcoma and a high-grade ovarian sex cord-stromal tumor (HG-SCST) with heterologous rhabdomyosarcomatous differentiation are reported. Clinical data were collected from medical records. Molecular profiling was unavailable at the time of diagnosis and treatment, and the tumor tissues were retrospectively analyzed. Representative archived formalin-fixed, paraffin-embedded (FFPE) blocks from both tumors, and paired nontumoral renal tissue, serving as a germline control for the renal tumor, were retrieved. Five FFPE sections (5–6 μm in thickness) were obtained from each selected block, and DNA was extracted using the QIAamp FFPE Tissue Kit (Qiagen, Hilden, Germany). DNA libraries were prepared with the AmpliSeq for Illumina Cancer HotSpot Panel v2 (Illumina, San Diego, CA, USA) targeting 2800 known somatic mutations across 50 clinically relevant genes [Supplementary file 1], and sequenced on the Illumina iSeq 100 platform (Illumina, San Diego, CA, USA). Data analysis and clinical interpretation were performed using the Illumina Connected Insights platform (https://www.illumina.com/). Variant pathogenicity was assessed using ten bioinformatic prediction (SIFT, PolyPhen-2, FATHMM-XF, AlphaMissense, REVEL, ClinPred, MetaSVM, MetaLR, MISTIC, and PrimateAI-3D), and the final curated variants were validated by Sanger sequencing and immunohistochemistry.

The first patient was a male born to consanguineous parents. He had a family history of XPC, including two maternal cousins and a younger brother, and had no reported history of internal malignancies. Genetic testing revealed a homozygous *XPC* mutation (NM_004628.5:c.850G>T [p.Glu284∗]). The patient had been protected from ultraviolet light (UV) since early childhood (UV-protective helmets and UV-filtering window films) and had no history of cutaneous malignancy. No ocular or neurological involvement was observed; however, mild but persistent erythema was noted after minimal sun exposure in his early childhood. At 14 years of age, he presented to the urology department with a four-week history of abdominal pain and gross hematuria. Abdominal ultrasound revealed a 9 cm solid-cystic mass in the upper pole of the right kidney. Thoraco-abdomino-pelvic computed tomography (CT) revealed a 10 cm × 9 cm × 7 cm renal mass with heterogeneous enhancement invading the renal pelvis without adrenal involvement or distant metastasis. A total ureteronephrectomy was performed.

**Figure 1:**
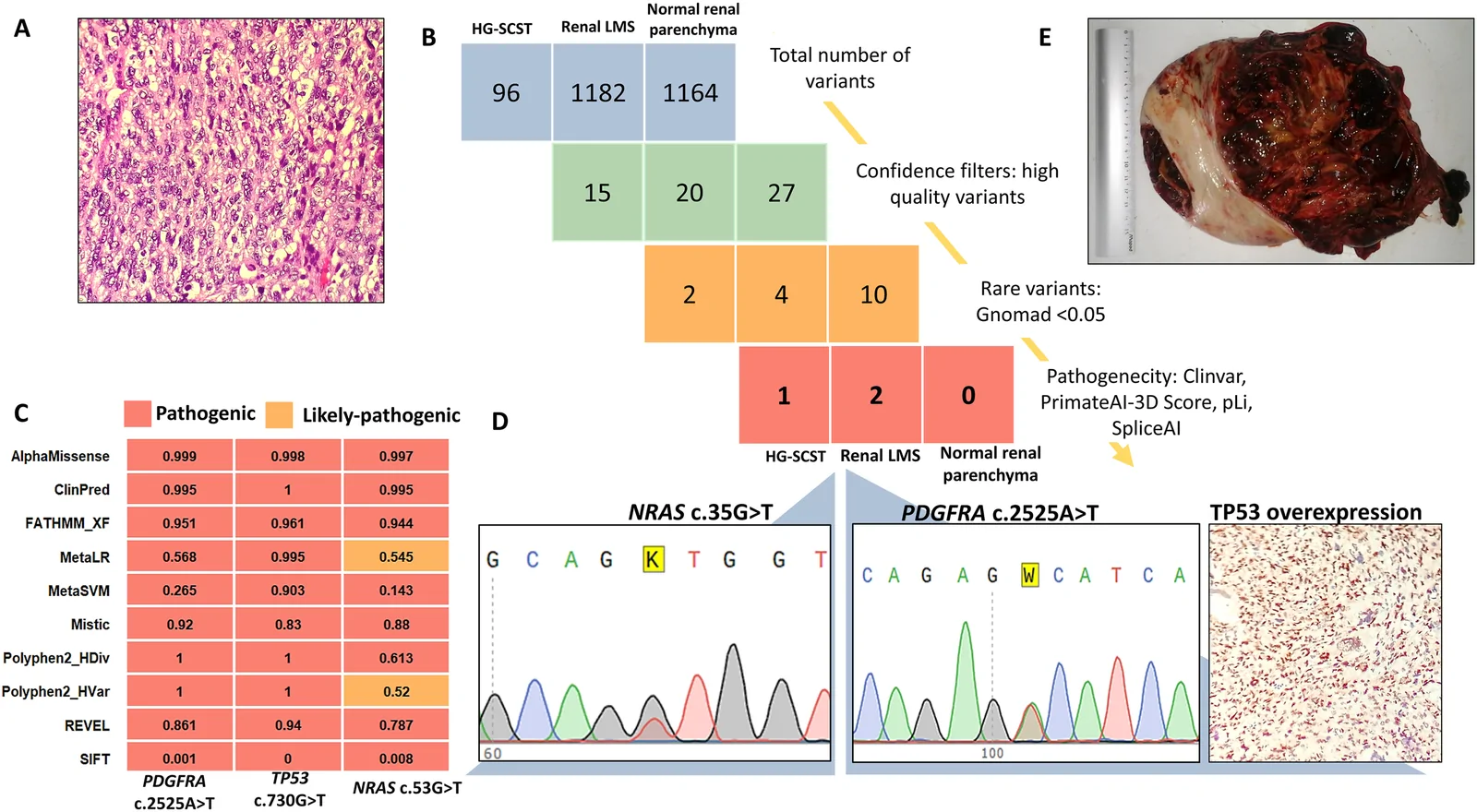
Histological and molecular characterization of renal leiomyosarcoma and ovarian HG-SCST in XPC patients. (A) Hematoxylin–Eosin-stained microphotograph (original magnification, × 100) of renal leiomyosarcoma showing spindle cells organized in long fascicles. (B) Variant filtration workflow showing total variant counts and progressive filtering across the three samples. (C) Computational pathogenicity prediction using 10 bioinformatic tools demonstrating the deleterious effects of filtered variants and prediction concordance across tools. (D) Validation of selected variants via Sanger sequencing for *PDGFRA* and *NRAS* mutations and immunohistochemical staining of p53 (original magnification, × 200). (E) Gross specimen of the resected ovarian mass showing a heterogeneous tumor with extensive hemorrhage and necrosis. HG-SCST: High-grade sex cord-stromal tumor; LMS: Leiomyosarcoma; *NRAS*: NRAS proto-oncogene, GTPase; *PDGFRA*: Platelet-derived growth factor receptor alpha; *TP53*: Tumor protein p53.

Histologically, the tumor was composed of atypical and highly mitotic spindle and pleomorphic cells [Figure 1A]. Immunohistochemistry showed diffuse positivity for smooth muscle actin, desmin, and h-caldesmon, with negativity for cytokeratin, WT1 transcription factor (WT1), human melanoma black-45 (HMB45), and Melan-A, consistent with a diagnosis of high-grade renal leiomyosarcoma. No postoperative complications were reported, and the patient received five cycles of adjuvant chemotherapy (vincristine, doxorubicin, cyclophosphamide, ifosfamide, and etoposide) at standard doses. No increased toxicity compared with the general population was reported.

After five years and four months of follow-up, he developed hepatic metastases and a locoregional recurrence that required surgical intervention. Two months later, rapid cardiac and renal failure occurred, and the patient died five years and eight months after diagnosis.

Targeted next-generation sequencing (NGS) identified 1182 variants in the renal leiomyosarcoma and 1,164 variants in the paired nontumoral tissue [Figure 1B]. Among them, two were pathogenic: a missense somatic mutation in exon 18 of platelet-derived growth factor receptor alpha (*PDGFRA*) (NM_006206.6:c.2525A>T (p.Asp842Val)) with a variant allele frequency (VAF) of 61.6% and a PrimateAI-3D score of 0.98, and a missense mutation in exon 7 of tumor protein p53 (*TP53*) (NM_000546.6:c.730G>T [p.Gly244Cys]) with a VAF of 39.9% and a PrimateAI-3D score of 0.94. Both mutations were absent in the paired nontumoral renal tissue and were classified as pathogenic by all ten bioinformatic prediction tools used [Figure 1C].

The identification of the activating *PDGFRA* p.Asp842Val mutation, which is typically associated with gastrointestinal stromal tumors (GISTs), raised the possibility of a misclassified extra-GIST. Additional immunohistochemical tests were therefore performed on the tumor. Cluster of differentiation (CD) 117 antigen was weakly expressed in rare tumor cells. Discovered on GIST-1 (DOG1) and CD34 antigens were negative. This profile, in conjunction with the strong h-caldesmon positivity, did not support the diagnosis of GIST, thereby favoring the diagnosis of leiomyosarcoma. Moreover, the tumor cells exhibited diffuse and strong nuclear p53 immunoreactivity, consistent with a *TP53-*mutated phenotype [Figure 1D]. This patient was previously described by Boulma et al., 2021.^2^ In the present report, we provide a five-year follow-up and complementary immunohistochemical and molecular analyses that were not available at the time of the original publication.

The second patient was an 18-year-old female, born to consanguineous parents, and under regular follow-up for XP since the age of seven. Genetic testing revealed a homozygous NM_004628.5:c.1643_1644delTG (p.Val548Alafs∗25) mutation in the *XPC* gene. She had a younger brother affected by XP. Her mother, who did not have XP, had a history of breast cancer.

The patient presented with a 15-day history of abdominal pain and constipation, along with one year of secondary amenorrhea. Physical examination revealed abdominal distension, diffuse tenderness, and tympanic note on percussion. Abdominopelvic CT revealed a large left ovarian solid-cystic mass. Surgical exploration revealed a 19 cm left ovarian solid-cystic tumor adhering to the bladder and pelvic wall, with multiple peritoneal implants [Figure 1E]. An incomplete tumor resection was performed.

Histopathological analysis established the definitive diagnosis of HG-SCST with heterologous rhabdomyosarcomatous differentiation. The patient received five cycles of chemotherapy at reduced doses consisting of weekly paclitaxel (60 mg/m^2^) and carboplatin (area under the curve [AUC] = 2 mg·min^-1^·mL^-1^). After three cycles, clinical evaluation showed disease stabilization with radiological stability and a decrease in the serum CA125 level (149 vs. 216 U/mL). However, by the fifth cycle, the patient experienced rapid disease progression with massive ascites and respiratory distress, and died one month after hospitalization.

Targeted NGS analysis of the tumor revealed 96 variants [Figure 1B]. After applying quality, frequency and pathogenicity filters, a single pathogenic variant was identified: a missense variant in exon 2 of the NRAS proto-oncogene, GTPase (*NRAS*) gene (NM_002524.5:c.35G>T (p.Gly12Val)), with a VAF of 50.2% and a PrimateAI-3D score of 0.96. It was classified as pathogenic by eight of the ten bioinformatics prediction tools used and likely pathogenic by the two others [Figure 1C] and was further confirmed by Sanger sequencing [Figure 1D].

## Discussion

The present study reports the clinical, pathological, and molecular characteristics of two rare internal tumors identified in XPC patients: a renal leiomyosarcoma and an ovarian HG-SCST with heterologous rhabdomyosarcomatous elements. These tumors represent highly uncommon histological variants at their respective anatomical sites. Renal leiomyosarcomas account for less than 1% of adult renal malignancies and are exceedingly uncommon in pediatric patients, and SCSTs represent approximately 8% of all primary ovarian neoplasms, with the majority being low-grade. To our knowledge, only seven cases of malignant ovarian tumors have been reported in patients with XP,^1^^,^^3^, ^4^, ^5^, ^6^ four of which were classified as SCSTs.^3^^,^^5^ Similarly, seven malignant renal tumors have been documented in XP patients. ^2^^,^^7^, ^8^, ^9^, ^10^, ^11^ Our case is the second reported renal leiomyosarcoma in this population and the first to include somatic mutational analysis of this tumor type in the context of XP [Table 1].

**Table 1:** Clinical and molecular features of renal and ovarian tumors in xeroderma pigmentosum patients.

Cancer site	Tumor type	Country	Age at tumor diagnosis (years)	Age at death (years)	Germline mutation	Somatic mutations	References
Ovary	High-grade serous carcinoma	Brazil	27	_	*XPC:* NM_004628.5: c.1643_1644delTG(p.Val548Alafs∗25)	_	Santiago et al., 2020^4^
	Two independent tumors in the same patient: juvenile granulosa-cell tumor (left ovary) and Sertoli-Leydig cell tumor (right ovary)	Algeria	19	_	*XPC:* NM_004628.5: c.1643_1644delTG (p.Val548Alafs∗25)	Juvenile granulosa-cell tumor: likely pathogenic missense and nonsense mutations in *DICER1* and an activating mutation in the *TERT* promoter Sertoli-Leydig cell tumor: likely pathogenic stop codon gain in *DICER1* and missense mutation in *TP53*^a^	Yurchenko et al., 2023^3^
	Poorly differentiated Sertoli-Leydig cell tumor with heterologous component and retiform pattern	Tunisia	11	_	*XPC:* NM_004628.5: c.1643_1644delTG (p.Val548Alafs∗25) *XPC:* NM_004628.5: c.850G>T (p.Glu284∗)	Missense and frameshift mutations in *DICER1*	Yurchenko et al., 2023^3^
	Mature cystic teratoma	China	29	_	*XPC:* NM_004628.5: c.2218_2220delCTC (p.Glu740_740del) *XPC*: NM_004628.5: c.2257dupC (p.Arg753fs)	_	Tang et al., 2018^6^
	Sarcoma	Morocco	18	22	*XPC*: NM_004628.5: c.1643_1644delTG (p.Val548Alafs∗25)	_	Nikolaev et al., 2022^1^
	Sertoli-Leydig tumor with rhabdomyomatous differentiation	Tunisia	27	_	_	_	Bdioui et al., 2021^5^
	HG-SCST with rhabdomyosarcomatous contingent heterologous	Tunisia	18	19	*XPC*: NM_004628.5: c.1643_1644delTG (p.Val548Alafs∗25)	*NRAS:* NM_002524.5: c.35G>T (p.Gly12Val)^b^	This study
Kidney	Rhabdoid tumor	Morocco	7 (F)	8	_	_	Ahsayen et al., 2022^8^
	Wilms tumor	Morocco	5 (M)	_	_	_	Lahlimi et al., 2016^11^
	Renal leiomyosarcoma	Spain	12 (F)	13	_	_	Tomás et al., 1989^9^
	Wilms tumor	Libya	17 (F)	18	_	_	Visweswara et al., 1997^10^
	Wilms tumor	Libya	16 (F)	17	_	_	Visweswara et al., 1997^10^
	Renal adenocarcinoma	Morocco	25 (F)	_	*XPC:* NM_004628.5:c.1643_1644delTG (p.Val548Alafs∗25)	_	Hadj-Rabia et al., 2013^7^
	Renal leiomyosarcoma	Tunisia	14 (M)	19	*XPC:* NM_004628.5: c.850G>T (p.Glu284∗)	*PDGFRA*: NM_006206.6: c.2525A>T (p.Asp842Val) *TP53:* NM_000546.6: c.730G>T (p.Gly244Cys)^b^	This study and Boulma et al., 2021^2^

*DICER1:* Dicer 1, ribonuclease III; HG-SCST: High-grade sex cord-stromal tumor; *NRAS*: NRAS proto-oncogene, GTPase; *PDGFRA*: Platelet-derived growth factor receptor alpha; *TERT*: Telomerase reverse transcriptase; *TP53*: Tumor protein p53; *XPC:* Xeroderma pigmentosum complementation group C; -: Data not available.

a Revealed by whole-genome sequencing.

b Targeted sequencing using AmpliSeq for Illumina Cancer HotSpot Panel.

The two patients reported in this study harbored germline mutations in the *XPC* gene. The *XPC* (p.Glu284∗) mutation identified in the first patient is a founder variant previously reported in only four unrelated Tunisian XP patients, including our patient.^12^^,^^13^ Three of these four patients developed internal malignancies, including a Sertoli-Leydig cell tumor, a thyroid carcinoma, and the renal leiomyosarcoma reported in this study, suggesting that this mutation may confer an increased risk of internal tumors. The *XPC* c.1643_1644delTG is a well-characterized founder mutation in North African XPC patients, particularly in the Maghreb region, where it accounts for 74% of affected families.^14^ In the French cohort of XPC patients, this mutation was associated with a 1300-fold increase in internal malignancies risk in young patients (0–20 years) and a 135-fold increase in female gynecological tumors.^1^ Beyond solid tumors, this mutation has also been linked to an increased risk of hematologic malignancies, including early-onset leukemias and myelodysplastic syndromes.^15^

In this study, targeted NGS revealed two pathogenic variants in the renal leiomyosarcoma: *TP53* p.Gly244Cys and *PDGFRA* p.Asp842Val. In response to DNA damage, p53 upregulates *XPC* transcription, and XPC affects p53 stability and activity, forming a feedback loop essential for genomic surveillance. Combined dysfunction of XPC and p53 results in a severely impaired cellular response to DNA damage, leading to heightened genomic instability and consequently a higher risk of cancer. The *TP53*-mutated status identified in the renal leiomyosarcoma in this study aligns with findings from sporadic leiomyosarcomas across various locations, which exhibit recurrent alterations in key tumor suppressor genes, including *TP53* [Supplementary Figure 1]. *PDGFRA* p.Asp842Val mutation is strongly associated with GISTs and rarely detected in leiomyosarcoma. It is known to confer primary resistance to first-line tyrosine kinase inhibitors (TKIs); however, next-generation TKIs have demonstrated efficacy against GISTs carrying this mutation and may represent a potential therapeutic option for other tumor types harboring this alteration. Interestingly, a sporadic case of abdominal leiomyosarcoma harboring concurrent variants in *PDGFRA* and *TP53,* similar to our case, was reported to respond to avapritinib.^16^ Unfortunately, tumor molecular profiling in our case was not available to guide clinical decision-making at the time of treatment.

The HG-SCST reported in this study revealed a well-characterized activating mutation in *NRAS* (c.35G>T (p.Gly12Val)), a classic hotspot mutation located at codon 12 in the GTPase domain. This alteration leads to persistent stimulation of downstream mitogen-activated protein kinase/extracellular signal-regulated kinase (MAPK/ERK) and phosphoinositide 3-kinase/AKT serine/threonine kinase (PI3K/AKT) pathways, suggesting potential therapeutic implications for XP patients. An analysis of three XP-associated ovarian SCSTs has shown somatic alterations in Dicer 1, ribonuclease III (*DICER1*), telomerase reverse transcriptase (*TERT*), and *TP53* [Table 1].^3^ *DICER1* and *TERT* were not included in the sequencing panel used in this study, and no pathogenic variants in the *TP53* gene were detected in our case. Germline *DICER1* mutations are known to be associated with a high risk for SCSTs, and somatic mutations in this gene are known drivers of these tumors. Recently, *DICER1* mutations were shown to occur exclusively in intermediate or poorly differentiated sporadic Sertoli-Leydig cell tumors and to be associated with retiform components, cords, or ribbon-like arrangements, and an earlier age of onset.^17^

Although our patients were effectively protected from UV-induced skin cancers, both developed fatal internal malignancies, underscoring the need to screen for extracutaneous tumors in XP patients. However, due to their rarity, no standardized consensus guidelines are currently available for the surveillance and management of these tumors. Periodic blood tests, clinical evaluations, and radiological examinations starting in childhood should be considered to detect these cancers at an early stage.

In conclusion, this study describes the clinical, pathological, and molecular features of a renal leiomyosarcoma and an HG-SCST in two XPC patients. While characterized by early onset and atypical histological features compared with their sporadic counterparts, these tumors carry well-known cancer driver mutations. These findings underscore the importance of proactive surveillance for extracutaneous malignancies in XP patients and highlight the potential benefit of targeted therapies in this vulnerable population with limited therapeutic options.

## Authors contribution

Soumaya Rammeh contributed to conceptualization, data curation, formal analysis, investigation, methodology, project administration, resources, validation, and writing of the original draft; Yasmine Ben Taher contributed to data curation, formal analysis, investigation, methodology and writing of the original draft; Mariem Ben Rekaya contributed to conceptualization, data curation, formal analysis, investigation, methodology, resources, and validation; Sarra Ben Rejeb, Faten Zeglaoui, and Meriem Jones contributed to conceptualization and validation; Neila Belguith contributed to conceptualization, methodology, project administration, validation, and review and editing. All the authors critically revised and approved the final paper.

## Ethics approval

This study was approved by the medical ethics committee of Charles Nicolle Hospital of Tunis (Approval No. ETHIQUE–HCN–011) and received exemption from patient consent.

## Data availability statement

The datasets used in the current study are available from the corresponding author on reasonable request.

## Declaration of Generative AI and AI-assisted technologies in the writing process

Artificial intelligence (AI) tools were utilized to enhance the readability and language of the manuscript. Following the application of this tool, the author reviewed and edited the content as appropriate and assumes full responsibility for all aspects of the publication.

## Funding

None.

## Conflict of interest

The authors declare that they have no known competing financial interests or personal relationships that could have appeared to influence the work reported in this paper.
