# Supplementary material for: Rare internal malignancies in xeroderma pigmentosum: A report of two cases from Tunisia and analysis of driver mutations
Source: Cancer Pathog Ther. 2026 Jan 21;4(4):326–30. doi: 10.1016/j.cpt.2026.01.003 (PMC13022642; doi:10.1016/j.cpt.2026.01.003)
Supplement: Multimedia component 1 [file mmc1.docx]

Supplementary Figure 1: Mutation profiles in sporadic leiomyosarcomas derived from The Cancer Genome Atlas (TCGA) (A) , and sporadic ovarian sex cord-stromal tumors derived from the Catalogue Of Somatic Mutations In Cancer (COSMIC) (B), compared with xeroderma pigmentosum-associated tumors from six published cases (C).


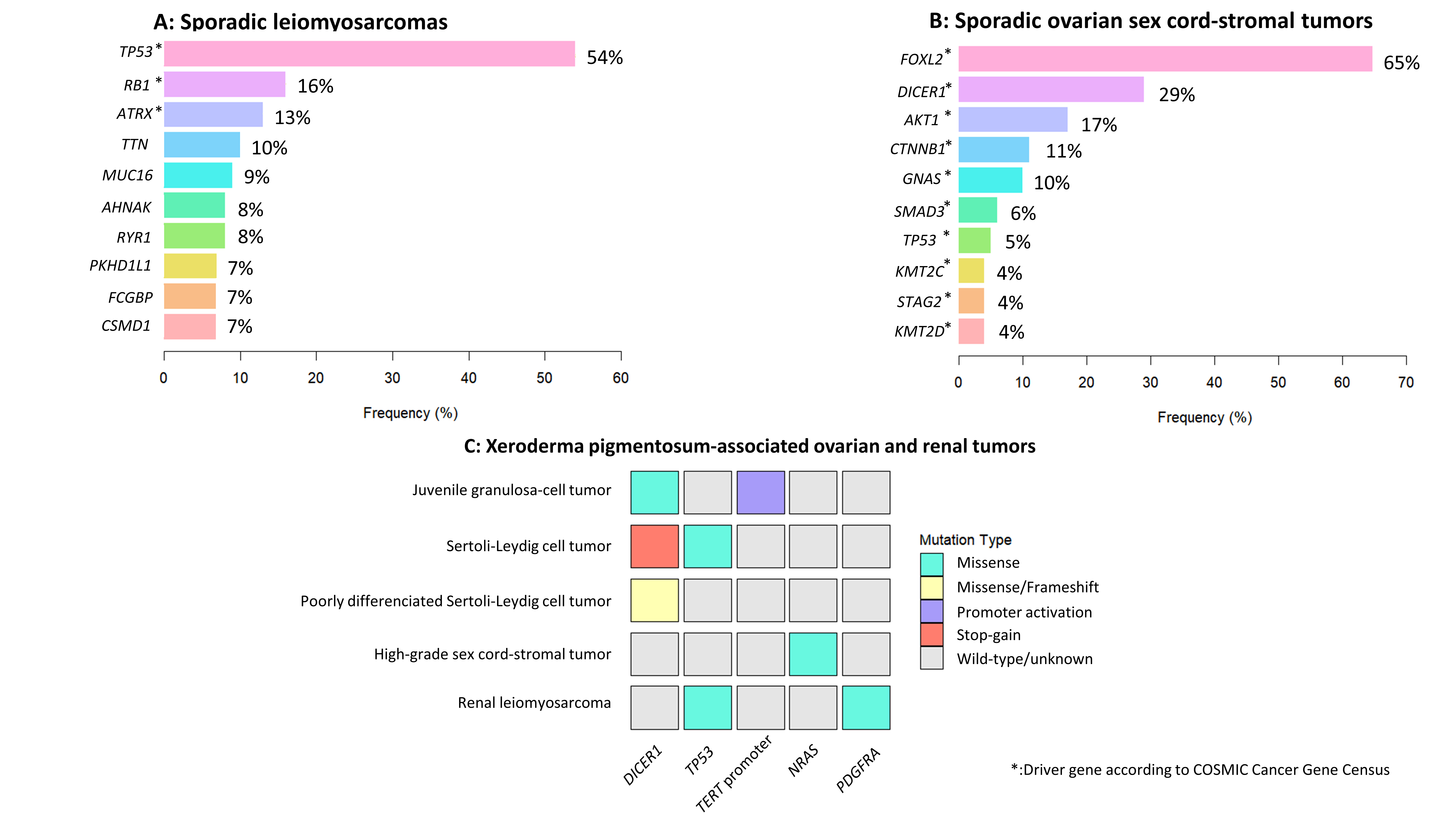


**Supplementary File 1. AmpliSeq Cancer HotSpot Panel v2 gene list**

- ***ABL1***: ABL proto-oncogene 1, non-receptor tyrosine kinase
- ***AKT1***: AKT serine/threonine kinase 1
- ***ALK****:* Anaplastic lymphoma receptor tyrosine kinase
- ***APC***: APC regulator of WNT signaling pathway
- ***ATM****:* ATM serine/threonine kinase
- ***BRAF***: B-Raf proto-oncogene, serine/threonine kinase
- ***CDH1***: Cadherin 1
- ***CDKN2A***: Cyclin dependent kinase inhibitor 2A
- ***CSF1R***: Colony stimulating factor 1 receptor
- ***CTNNB1***: Catenin beta 1
- ***EGFR***: Epidermal growth factor receptor
- ***ERBB2****:* Erb-b2 receptor tyrosine kinase 2
- ***ERBB4****:* Erb-b2 receptor tyrosine kinase 4
- ***EZH2***: Enhancer of zeste 2 polycomb repressive complex 2 subunit
- ***FBXW7***: F-box and WD repeat domain containing 7
- ***FGFR1***: Fibroblast growth factor receptor 1
- ***FGFR2****:* Fibroblast growth factor receptor 2
- ***FGFR3****:* Fibroblast growth factor receptor 3
- ***FLT3***: Fms related receptor tyrosine kinase 3
- ***GNA11***: G protein subunit alpha 11
- ***GNAS****:* GNAS complex locus
- ***GNAQ***: G protein subunit alpha q
- ***HNF1A****:* HNF1 homeobox A
- ***HRAS***: HRas proto-oncogene, GTPase
- ***IDH1***: Isocitrate dehydrogenase (NADP(+)) 1
- ***IDH2***: Isocitrate dehydrogenase (NADP(+)) 2
- ***JAK2***: Janus kinase 2
- ***JAK3****:* Janus kinase 3
- ***KDR***: Kinase insert domain receptor
- ***KIT***: KIT proto-oncogene, receptor tyrosine kinase
- ***KRAS***: KRAS proto-oncogene, GTPase
- ***MET***: MET proto-oncogene, receptor tyrosine kinase
- ***MLH1***: MutL homolog 1
- ***MPL****:* MPL proto-oncogene, thrombopoietin receptor
- ***NOTCH1***: Notch receptor 1
- ***NPM1***: Nucleophosmin 1
- ***NRAS***: NRAS proto-oncogene, GTPase
- ***PDGFRA***: Platelet derived growth factor receptor alpha
- ***PIK3CA***: Phosphatidylinositol-4,5-bisphosphate 3-kinase catalytic subunit alpha
- ***PTEN***: Phosphatase and tensin homolog
- ***PTPN11***: Protein tyrosine phosphatase non-receptor type 11
- ***RB1***: RB transcriptional corepressor 1
- ***RET***: RET proto-oncogene, receptor tyrosine kinase
- ***SMAD4***: SMAD family member 4
- ***SMARCB1***: SWI/SNF related, matrix associated, actin dependent regulator of chromatin, subfamily b, member 1
- ***SMO***: Smoothened, frizzled class receptor
- ***SRC***: SRC proto-oncogene, non-receptor tyrosine kinase
- ***STK11***: Serine/threonine kinase 11
- ***TP53***: Tumor protein p53
- ***VHL***: Von Hippel–Lindau tumor suppressor
